# Morphological heterogeneities in prostate cancer bone metastases are related to molecular subtypes and prognosis

**DOI:** 10.1007/s10585-025-10365-y

**Published:** 2025-08-21

**Authors:** Sofia Halin Bergström, Julius Semenas, Annika Nordstrand, Elin Thysell, Johan Wänman, Sead Crnalic, Anders Widmark, Camilla Thellenberg-Karlsson, Pernilla Andersson, Susanne Gidlund, Marie Lundholm, Karin Welén, Andreas Josefsson, Pernilla Wikström, Anders Bergh

**Affiliations:** 1 Department of Medical Biosciences, Pathology, Umeå University, Umeå, Sweden; 2 Department of Diagnostics and Interventions, Orthopedics, Umeå University, Umeå, Sweden; 3 Department of Diagnostics and Interventions, Oncology, Umeå University, Umeå, Sweden; 4 Department of Urology, Sahlgrenska Centre for Cancer Research, Institute of Clinical Sciences, Sahlgrenska Academy, University of Gothenburg, Gothenburg, Sweden; 5 Department of Diagnostics and Interventions, Urology and Andrology, Umeå University, Umeå, Sweden

**Keywords:** Prostate cancer metastases, Bone metastasis stroma, Metastatic stroma, Prostate cancer molecular subtypes, Tumor heterogeneity, Stromal markers, Metastases morphology, Tumor microenvironment, Androgen deprivation therapy

## Abstract

We previously identified three molecular subtypes of prostate cancer (PC) bone metastases, MetA-C, with MetB linked to poor prognosis after androgen deprivation therapy (ADT). This study analyzed epithelial and stromal markers using immunohistochemistry, focusing on their relationship to MetA-C subtypes, spatial heterogeneities, and clinical outcomes after ADT. High tumor proliferation and low PSA expression were associated with MetB and poor outcomes after ADT. Most metastases contained tumor epithelial subclones with different morphologies. In the metastasis stroma, blood vessels and fibroblast-like cells expressed smooth muscle actin (SMA), platelet-derived growth factor β, stroma-derived factor 1 (SDF1), periostin (POSTN), and decorin (DCN). Compared to each other, MetB metastases had higher SMA and ERG + endothelial cell densities, while MetA cases showed higher SDF1 and DCN levels. Accordingly, high POSTN and ERG + densities were associated with poor outcomes after ADT, whereas high DCN indicated favorable prognosis. Low levels of AR-positive stromal cells were linked to poor outcomes. Macrophage and T-lymphocyte densities showed no significant associations with metastases subtypes or outcome. Two stroma subtypes were identified: subtype 1 with higher bone content, lower vessel density, MetA-enrichment and better prognosis compared to subtype 2 that exhibited higher tumor proliferation and lower PSA expression. Most metastases contained regions of both stroma subtypes.

**Supplementary Information:**

The online version contains supplementary material available at 10.1007/s10585-025-10365-y.

## Introduction

Cancers confined to the prostate can be cured with surgery or radiotherapy. However, the presence of clinically detectable bone metastases often indicates a lethal stage of the disease. Current treatments for bone metastatic prostate cancer (PC), which target androgen synthesis (androgen deprivation therapy, ADT) and/or androgen receptor (AR) signaling, along with taxanes, provide only temporary relief [1], highlighting the need for more effective treatments. Clinical observations and experimental studies suggest that tumor response to ADT is stronger in the prostate than in the bone [2–4]. However, the underlying reasons for this differential response remain poorly understood. Further research is needed to explore the insufficient ADT response in metastases and the factors involved, including the role of the metastatic microenvironment.

The global transcriptome, proteome, and morphology show distinct differences between primary tumors and metastases [5–8]. Additionally, PC bone metastases can be divided into subgroups based on biological characteristics, morphology and clinical outcomes [5–7, 9–18]. Based on transcriptomic differences, PC bone metastases can be classified into three subgroups: MetA, MetB, and MetC [6, 7]. MetA (~ 70% of cases) shows high expression of androgen-regulated genes, including KLK3 (PSA), and MetA patients have relatively good prognosis after ADT and AR-targeted therapies. MetB (~ 20% of cases), the most aggressive subtype, is characterized by high cell proliferation, a dedifferentiated luminal cell phenotype, low PSA, and poor prognosis after ADT. MetC, a small subgroup (~ 10% of cases), is associated with high stroma-related gene expression, low PSA, and intermediate prognosis.

The distinct gene expression patterns of the MetA-C subtypes suggest that different mechanisms, including varying stroma-epithelial interactions, might drive their behavior, such as treatment response, disease progression, and overall prognosis [6, 7]. Stroma-epithelial interactions are known to influence primary PC growth and treatment response [19–24]. However, the relationship between attenuated efficacy of ADT therapy in bone metastases and the specific cell types present in the bone metastasis microenvironment [25, 26] remains underexplored.

The most contrasting metastasis subtypes, MetA and MetB, can be identified using two immunohistochemical markers: Ki67 for tumor cell proliferation and PSA for luminal cell differentiation and androgen receptor (AR) activity [7, 8]. High Ki67 and low PSA levels indicate a dominating MetB signature, while MetA metastases show the opposite pattern [7]. Gene expression analysis also suggests that most bone metastases are heterogeneous for MetA-C subtypes and that increased fraction of cells with MetB characteristics is a strong negative prognostic factor [6]. This raises the possibility that spatial variations in metastasis morphology and ADT response exist.

This study aimed to characterize morphological heterogeneities in the epithelial and stromal compartments of human PC bone metastases and to investigate their relationship with the transcriptomic subtypes MetA-C and patient outcome following ADT. We quantified immunohistochemical markers specific to epithelial and stromal cells, assessed bone content and overall stroma morphology, and evaluated marker heterogeneity within metastasis samples. Furthermore, we analyzed the associations between epithelial and stromal markers, morphology, MetA-C subtypes, and outcomes following ADT. Lastly, we compared the expression levels of these markers across three patient groups: untreated patients, those on short-term ADT, and patients with CRPC.

## Materials and methods

### Patients

The study includes 167 patients with metastatic PC from whom metastatic tissue samples were available (Table 1). In most cases, the metastatic tissue was obtained during surgery due to metastatic spinal cord compression (*n* = 138) or pathological hip fractures (*n* = 13) at Umeå University Hospital or at the Sahlgrenska University Hospital between 2003 and 2021. In other cases, core biopsies were obtained during treatment for castration-resistant PC (CRPC) from the iliac crest (*n* = 15) or rib (*n* = 1) metastases. At the time of sampling, patients were either hormone-naïve (*n* = 30), castration-resistant (*n* = 121), or treated with ADT for a short period ranging between 1 and 22 days (short-term castrated, *n* = 16). All patients gave their informed consent, and the study was conducted in accordance with the Declaration of Helsinki. The study was approved by the local ethic review boards of Umeå University (Dnr 03-158 with amendments Dnr 04-26 M, Dnr 2013-57-31 M) and Gothenburg (Dnr 455 − 11 with amendment 2019–05732).

**Table 1:** Clinical characteristics of 167 patients with bone metastasis tissue samples explored for morphological markers

	Median (25th; 75th percentiles)
Age diagnosis (yrs.)	70 (65; 76)
Age metastasis sampling (yrs.)	73 (68; 78)
Serum PSA diagnosis (ng/ml)	91 (26; 700)
Serum PSA metastasis sampling (ng/ml)	160 (44; 800)
Follow-up from diagnosis (yrs.)	3.9 (2.0–7.2)
Follow-up from ADT (yrs.)	3.5 (2.0–6.4)
Follow-up from metastasis surgery (yrs.)	1.0 (0.32–2.6)

Metastasis site	N (%)
Spine	138 (83)
Iliac crest	15 (9.0)
Hip	13 (7.8)
Rib	1 (0.6)
Castration therapy^a^	
None (hormone-naïve)	30 (18%)
Short-term^b^	16 (9.6%)
Long-term	121 (73%)
Treatment for CRPC	
Palliative radiation (towards metastasis)	23/121
Bicalutamide	56/118
Chemotherapy	33/121
Abiraterone acetate	12/121
Enzalutamide	4/121
Ra223	3/121
Zoledronic acid/Denosumab	5/121

Continuous variables given as median (25th; 75th percentiles). ^a^Castration therapies given prior to collection of metastasis tissue samples included surgical ablation, LHRH/GnRH agonist therapy or bicalutamide treatment. ^b^Short-term treatment for 1–22 days before metastasis tissue sampling.

### Morphology and immunohistochemical analysis

Formalin-fixed paraffin-embedded (FFPE) metastasis tissue samples were stained with hematoxylin and eosin for morphological evaluation of growth pattern involving stromal subtype, bone (woven + lamellar) density, and apoptosis. Using the automated Ventana BenchMark Ultra platform (Ventana Medical systems), sections were stained for: PSA (Dako, #A0562, 1:1000), Ki67 (Ventana, #790–4286, clone 30 − 9, Ready-to-use (RTU)), AR (Biogenex, #MU256-UC, clone F39.4.1, 1:50), CHRA (Ventana, #760–2519, clone LK2H10, RTU), SMA (Dako, #M0851, clone 1A4, 1:150), POSTN (Atlas, #HPA012306, 1:100), DCN (Atlas, #HPA003315, 1:500), CD68 (Dako, #M0814, clone KP1, 1:2000), or CD3 (Ventana, #790–4341, clone 2GV6, RTU), with Ultra Cell conditioning 1 (CC1) as antigen retrieval buffer and developed with the UltraView Universal DAB Detection kit (PSA, Ki67, CHRA, SMA, POSTN, DCN, CD68) or the OptiView DAB IHC Detection kit (AR, CD3). ERG (Biocare Medical, #CM421A, clone 9FY, 1:50), PDGFRB (Cell Signaling, #3169S, clone 28E1, 1:75), and SDF1 (R&D Systems, MAB350, clone 79018, 1:50) were stained manually with antibodies incubated overnight at + 4 °C, Tris-EDTA pH 9 was used as antigen retrieval. The detection system MACH3 Mouse HRP-Polymer kit (Biocare Medical) was used for ERG and SDF1, and a biotinylated goat anti-rabbit and the Vectastain ABC-HRP kit (Vector Laboratories) were used for PDGFRB. All sections were developed with DAB.

The PSA and AR staining in tumor epithelial cells were quantified using a scoring system based on the percentage (0: 0%, 1: 1–25%, 2: 26–50%, 3: 51–75%, and 4: 76–100%) and intensity (0: negative, 1: week, 2: moderate, and 3: intense staining) of immunostained cells. An immunoreactivity (IR) score was obtained by multiplying the scores for distribution and intensity, giving IR scores in the range of 0–12. Ki67 and CHRA in tumor epithelial cells were quantified as the percentage of stained tumor cells, evaluated in 500–1000 cells per patient. Tumor cells were also scored as ERG positive or ERG negative. The volume density of SMA + stroma, PDGFRB + stroma, SDF-1 + stroma, POSTN + stroma, DCN + stroma, ERG + endothelial cells, CD68 + macrophages, and CD3 + T-lymphocytes, was quantified using a square-lattice mounted in the eyepiece of the microscope counting grid-intersection falling on the stained tissue component and on reference space. Similarly, the density of bone was quantified on hematoxylin–eosin-stained sections. The fraction of apoptotic epithelial cells was scored in at least 1000 cells per sample as earlier described [3]. The fraction of stroma cells positive for AR and for Ki67 was calculated after examining approximately 1000 stroma cells per sample.

Chromogenic double staining was performed on the automated Ventana Discovery Ultra platform (Ventana Medical systems), except for double staining with ERG. Here, automated staining was preceded by manually stained ERG (see above). Antibodies used were the same as for single staining (see above and Table 2 for more details). Ultra CC2 was used for antibody denaturation (100°C, 8′) and Discovery Inhibitor was used for blocking of endogenous peroxidase (8′). Hematoxylin II (4′) and Bluing (4′) were used as counterstain.

**Table 2:** Protocol parameters for discovery ultra chromogenic double-staining

	PSA/Ki67	ERG/PDGFRB	ERG/SMA	ERG/DCN	ERG/POSTN	ERG/SDF1	DCN/PDGFRB	DCN/SMA
Antibody (dilution, minutes)	PSA (1:1000, 32′)	ERG (manual staining)	ERG (manual staining)	ERG (manual staining)	ERG (manual staining)	ERG (manual staining)	PDGFRB (1:75, 60′)	SMA (1:100, 32′)
Antigen retrieval (temperature, minutes)	–	–	–	–	–	–	Ultra CC1 (95 °C, 64′)	Ultra CC1 (95 °C, 32′)
Multimer (minutes)	UMap anti-Rb AP (16′)	–	–	–	–	–	OMap anti-Rb HRP, (16′)	OMap anti-Ms HRP (16′)
Chromogen (minutes)	DISCOVERY Yellow (44′)	–	–	–	–	–	DISCOVERY Purple (32′)	DISCOVERY Purple (32′)
Antibody (dilution, minutes)	Ki67 (RTU, 32′)	PDGFRB (1:50, 60′)	SMA (1:100, 32′)	DCN (1:500, 32′)	POSTN (1:150, 32′)	SDF1 (1:50, 32′)	DCN (1:200, 32′)	DCN (1:200, 32′)
Antigen retrieval (temperature, minutes)	CC1 (95 °C, 64′)	–	–	–	–	–	–	Ultra CC1 (95 °C, 16′)
Multimer (minutes)	OMap anti-Rb HRP (16′)	OMap anti-Rb HRP, (16′)	OMap anti-Ms HRP, (16′)	OMap anti-Rb HRP, (16′)	OMap anti-Rb HRP, 16′	OMap anti-Ms HRP, 16′	OMap anti-Rb HRP, 20′	OMap anti-Rb HRP, 20′
Chromogen (minutes)	DISCOVERY Purple 32′	DISCOVERY Purple 32′	DISCOVERY Purple 32′	DISCOVERY Purple 32′	DISCOVERY Purple 32′	DISCOVERY Purple 32′	DISCOVERY Yellow HRP 32′ + 16′	DISCOVERY Yellow HRP 32′ + 16′

To perform multiplex immunofluorescence analysis, we used the Opal Fluorophore reagent packs (Akoya Biosciences) and the Discovery Ultra platform (Ventana Medical Systems). Slides were manually deparaffinized, rehydrated and pre-treated with Tris/EDTA pH 9. Sequential repetitive cycles of primary antibody, secondary HRP antibody followed by Opal Fluorophore were performed for each antibody; (1) SDF1 (1:25, 1 h; OMap-Ms, 16′; O-520, 1:50, 32′), (2) PDGFRB (1:75, 1 h; OMap-Rb, 16′; O-570, 1:100, 20′), (3) DCN (1:300, 1 h; OMap-Rb, 16′; O-620, 1:100, 20′), (4) SMA (1:100, 32′; OMap-Ms, 16′; O-650, 1:50, 20′), and (5) Pan-Cytokeratin AE1/AE3 (DAKO, #M3515) (1:50, 32′; OMap-Ms, 16′; O-690, 1:50, 20′). For antibody denaturation, Ultra CC2 was used between each cycle (100 °C, 8′). To quench endogenous peroxidase the first sequence was preceded by Discovery Inhibitor (8′). After the last cycle, 1x Spectral DAPI (Akoya Biosciences) was used as nuclear stain (5′).

### Classification of the metastasis subtypes MetA-C

For 119 of the metastasis cases, frozen tissue samples had been previously profiled by whole-genome transcriptomic analysis using either the human HT12 Illumina Beadchip technique (Illumina, San Diego, CA, USA) [10, 15] or the Clariom D Human Array technique (Thermo Fisher Scientific Inc., Life Technologies, Carlsbad, CA, USA [6].

Here, data sets (GSE29650, GSE101607, GSE189343, https://www.ncbi.nlm.nih.gov/geo/) were merged and batch-corrected to enable combined analysis. The fraction (0.0**–**1.0) of each tissue sample with MetA-C characteristics was determined based on the expression levels of 157 pre-defined MetA-C-differentiating genes, as previously described [6]. Furthermore, samples were classified into MetA, MetB or MetC based on their dominant subtype [6].

### Statistics

Continuous variables were given as median (25th; 75th percentiles) and ordinal variables were given as number (percentage). Non-parametric statistics were used to compare groups and for correlation analyses (Kruskal-Wallis test with Dunn’s multiple comparison test and the Spearman rank correlation test). Morphological variables were dichotomized as positive/negative or based on quartiles in survival analysis. Cancer-specific survival was analyzed using the log-rank test with death from PC as event and other causes as censored events. Follow-up started at the date of ADT and ended at the date of death or the latest follow-up. All tests were two sided and *p*-values less than 0.05 were considered statistically significant. Statistical analysis was performed using the GraphPad Prism version 10, and SPSS version 28.

## Results

### Tumor epithelial markers

#### PSA and Ki67

**Fig. 1:**
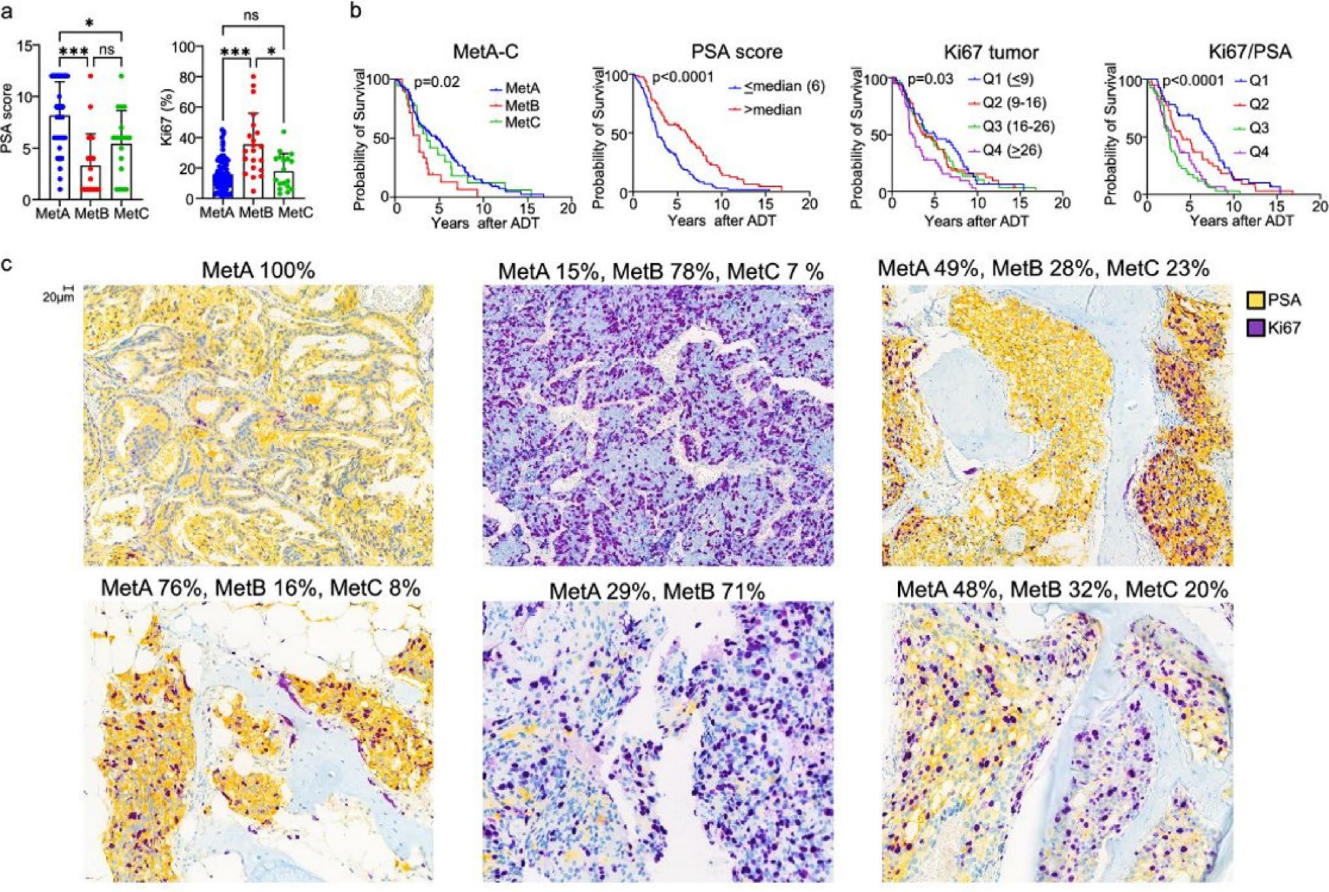
Tumor cell proliferation and PSA expression in relation to the molecular metastasis subtypes MetA-C and patient prognosis. **a** Graphs showing how tumor epithelial cell PSA and Ki67 staining scores differ between MetA, Met B and MetC dominating metastasis subtypes. * *p* < 0.05, *** *p* < 0.001, ns = not significantly different. **b** Kaplan-Meier curves showing survival after androgen-deprivation therapy (ADT) in relation to dominant molecular subtypes (MetA-C), tumor cell PSA, Ki67, and Ki67/PSA staining scores. **c** Sections from bone metastases, classified to contain different fractions of the MetA, MetB and MetC subtypes, double-stained for PSA and Ki67. Metastases with high MetA fractions (left) showed homogeneously strong PSA and low Ki67 staining, whereas metastases with high MetB fractions (middle) showed the opposite pattern. In metastases with a dominating MetA fraction but also containing a substantial MetB component (right), local areas with moderate to high PSA and low to moderate Ki67 (MetA-like pattern) and adjacent areas with low PSA and high Ki67 (MetB-like pattern) were seen, suggesting spatial heterogeneities in tumor cell phenotypes

PSA and proliferation (Ki67) immunostaining scores were measured as surrogate markers for the molecular MetA and MetB metastases subtypes and as markers linked to patient prognosis after ADT [6, 7]. In this metastasis cohort, the mean PSA staining score of tumor epithelial cells was 7 (median 6, Q1 4, Q4 10, range 1–12, *n* = 164) and the mean Ki67 + fraction was 20% (median 16, Q1 9, Q4 26, range 1–80, *n* = 162), indicating substantial variability between patients (Supplement Fig. 1). The transcriptomic MetA-C subtypes were evaluated in 119 cases, demonstrating mean MetA, B, and C fractions of 55, 24, and 21%, respectively (MetA: median 58, Q1 39, Q4 76, range 0-100, MetB: median 18, Q1 5, Q4 33, range 0-100, and MetC: median 15, Q1 3, Q4 29, range 0–95). Classified based on the dominant subtype of each sample, the cohort contained 80 MetA, 21 MetB, and 18 MetC patients. PSA and Ki67 scores were inversely correlated, with PSA significantly correlated to fraction of MetA and Ki67 to fraction of MetB (Supplement Fig. 2). Patients with MetA-dominant metastases had significantly higher PSA score and lower Ki67 levels compared to patients with MetB-dominant metastases (Fig. 1a). Consistent with prior findings, the MetB subtype, low PSA, high Ki67, and high Ki67/PSA ratio were associated with short survival after ADT (Fig. 1b).

**Fig. 2:**
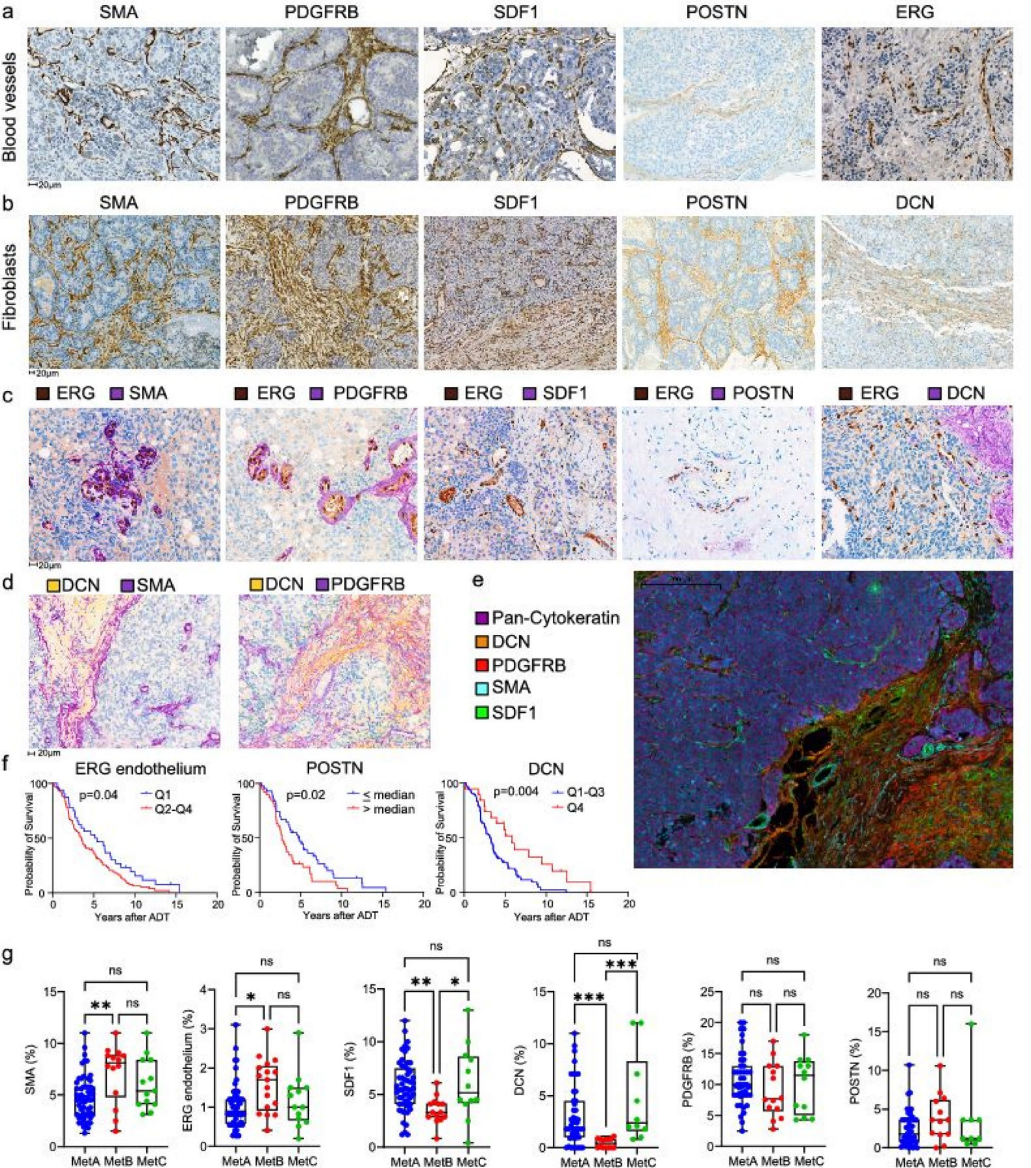
Stroma cell heterogeneities in relation to the molecular metastasis subtypes MetA-C and patient prognosis. Immunohistochemical localization of factors expressed in **a** blood vessel walls and **b** fibroblast-like cells. **c** Double staining of ERG (localized in endothelial cell nuclei) and various stroma cell markers. All markers except DCN were localized adjacent to endothelial cells. **d** Double staining of DCN + SMA, and DCN + PDGFRB. SMA was expressed mainly in blood vessel walls adjacent to tumor epithelial cells whereas DCN was expressed in the broad stroma septa. PBGDRB was expressed adjacent to DCN in broad stroma septa and in blood vessels adjacent to tumor cells. **e** Multiplex staining showing that most stroma cell markers were expressed in the broad stroma septa whereas SMA, SDF-1 and focal PDGFRB were also detected in and around the tiny blood vessels penetrating tumor cell nests. **f** Kaplan-Meier analysis showing that survival after androgen-deprivation therapy (ADT) was related to the density of ERG + endothelial cells and to the stroma densities of POSTN and DCN. **g** Boxplots showing densities of stroma SMA, ERG, SDF-1, DCN, PDGFRB, and POSTN in relation to dominating metastasis subtypes MetA-C. *p < 0.05, **p < 0.01, ***p < 0.001, ns = not significantly different

As most metastases consist of varying fractions of the MetA-C subtypes [6], individual metastases may be heterogeneous and contain tumor cell clones with different molecular profiles. To further investigate spatial heterogeneity, we performed double staining for PSA and Ki67 in cases with known MetA-C subtype fractions.

In cases with a high MetA fraction (Q4, MetA > 75%), tumor cells showed strong, homogeneous PSA staining, with relatively few Ki67 + cells, primarily located at periphery of tumor cell nests, resembling the pattern seen in normal prostate glands (Fig. 1c). In other cases, Ki67 + tumor cells were observed both in the central and peripheral parts of tumor cell nests (Fig. 1c). Minor areas within the same metastasis sometimes showed high Ki67 and low PSA expression, suggesting focal development of more malignant clones within MetA-dominant tumors (Fig. 1c).

In cases with a high MetB fraction (Q4, MetB > 33%), PSA staining was largely absent or low and scattered, while Ki67 + cells were abundant throughout the tumor (Fig. 1c). In metastases with mixed MetA and MetB fractions, PSA and Ki67 labeling varied, suggesting that different areas were enriched for either MetA (high PSA/low Ki67) or MetB (low PSA/high Ki67) (Fig. 1c) but areas with high Ki67 and high PSA could also be observed. The size of these areas varied between cases, where some areas were rather small (evident only at high magnification) whereas others were considerably larger (evident at low magnification) (Fig. 1c).

Overall, this suggests inter-patient and intra-patient variability in epithelial cell phenotypes.

#### Androgen receptor (AR), chromogranin A (CHRA), apoptosis and tumor ERG

The mean AR staining score in tumor epithelial cells was 8, but with large variability observed (median 8, Q1 4, Q4 12, range 0–12, *n* = 156) (Supplement Fig. 1). The neuroendocrine marker CHRA was undetectable in 94 of 139 samples with a mean CHRA + cell density of 2% (median 0, Q1 0, Q4 0.2, range 0–80%, *n* = 139) (Supplement Fig. 1). In cases with CHRA + tumor cells, positive cells were generally isolated, located next to unstained cells (Supplement Fig. 1). Tumor cell apoptosis was generally low with a mean apoptotic tumor cell density of 1% (median 0.5, Q1 0.3, Q4 1.0, range 0–23%, *n* = 78) (Supplement Fig. 1). Metastatic tumor cells were ERG + in 26% of the cases (36 of 139), suggesting TMPRSS2-ERG fusion gene expression [27] (Supplement Fig. 1).

The tumor cell AR score, CHRA levels, apoptosis levels, or ERG score were not associated with outcome, and did not differ between patients with different metastasis subtypes (MetA-C), except for CHRA that was significantly lower in MetC compared to MetB patients (Supplement Fig. 1).

### Tumor stroma markers

#### Markers of blood vessels and fibroblast-like cells

Staining of smooth muscle actin (SMA), platelet derived growth factor β (PDGFRB), stroma derived factor 1 (SDF1), periostin (POSTN), and ERG was observed in blood vessels within both normal bone marrow and bone metastases (Fig. 2a). SMA, PDGFRB and POSTN were detected in pericytes and vascular smooth muscle cells in both large and small vessels, endothelial cells stained positive for SDF1, POSTN, and ERG (Fig. 2a). In some cases, SMA and PDGFRB staining was focal, localized to the mural layer of small blood vessels penetrating tumor nests (Fig. 2a).

SMA, PDGFRB, SDF1, POSTN, and DCN were also found in fibroblast-like cells within the connective tissue septa separating tumor cell nests (Fig. 2b), with POSTN and DCN occasionally present at the bone surface (data not shown). Additionally, tumor cells stained positive for SDF1 in some patients (data not shown).

The mean densities of SMA + cells and SDF1 + stroma were 5% of the metastasis volume (SMA: median 5, Q1 3.1, Q4 6.9, range 0.7–13.0, *n* = 108; SDF1: median 5, Q1 3.5, Q4 6.8, range 0.4–13.0, *n* = 117), while PDGFRB + stromal cells had approximately double the density, in mean constituting 10% of the metastasis volume (median 10, Q1 6.6, Q4 13, range = 1.2–22.0, *n* = 91). POSTN + and DCN + cells comprised about 3% each of the metastasis volume (POSTN: median 2.3, Q1 0.82, Q4 3.6, range 0–16, *n* = 80; DCN: median 1.8, Q1 0.83, range 0–14, Q4 4.5, *n* = 84), while ERG + endothelial cells had a mean density of 1% (median 1.0, Q1 0.66, Q4 1.5, range 0.2–3.6, *n* = 127).

To investigate stromal cell types adjacent to endothelial cells, double staining for ERG and the other stromal markers was performed. ERG + nuclei were frequently found close to SDF1 + endothelial cells, particularly in the smallest blood vessels near tumor cell nests (Fig. 2c). ERG + cells were also found adjacent to SMA+, PDGFRB+, and POSTN + mural vascular cells (Fig. 2c). However, ERG + nuclei were rarely found near DCN + cells and extracellular matrix. DCN was instead located closer to PDGFRB + or SMA + cells within the broader connective tissue septa (Fig. 2d). Notably, DCN + cells were generally absent from the small PDGFRB+/SMA + vessels within the tumor cell nests (Fig. 2d). Multiplex staining confirmed that SMA, PDGFRB, SDF1, and DCN were highly expressed in the broader connective tissue septa (Fig. 2e). Within the tumor, SDF-1, SMA, and focal PDGFRB were observed in the thinner stroma strands and micro-vessels (Fig. 2e). The highest number of tumor cells in direct contact with stroma was found along these thin stroma septa and micro-vessels.

The densities of SMA+, PDGFRB+, or SDF1 + cells were not significantly related to ADT outcomes (Supplement Fig. 3). However, higher densities of ERG + endothelial cells or high POSTN levels were significantly associated with poor ADT outcomes (Fig. 2f). In contrast, high DCN levels were significantly associated with better prognosis (Fig. 2f).

**Fig. 3:**
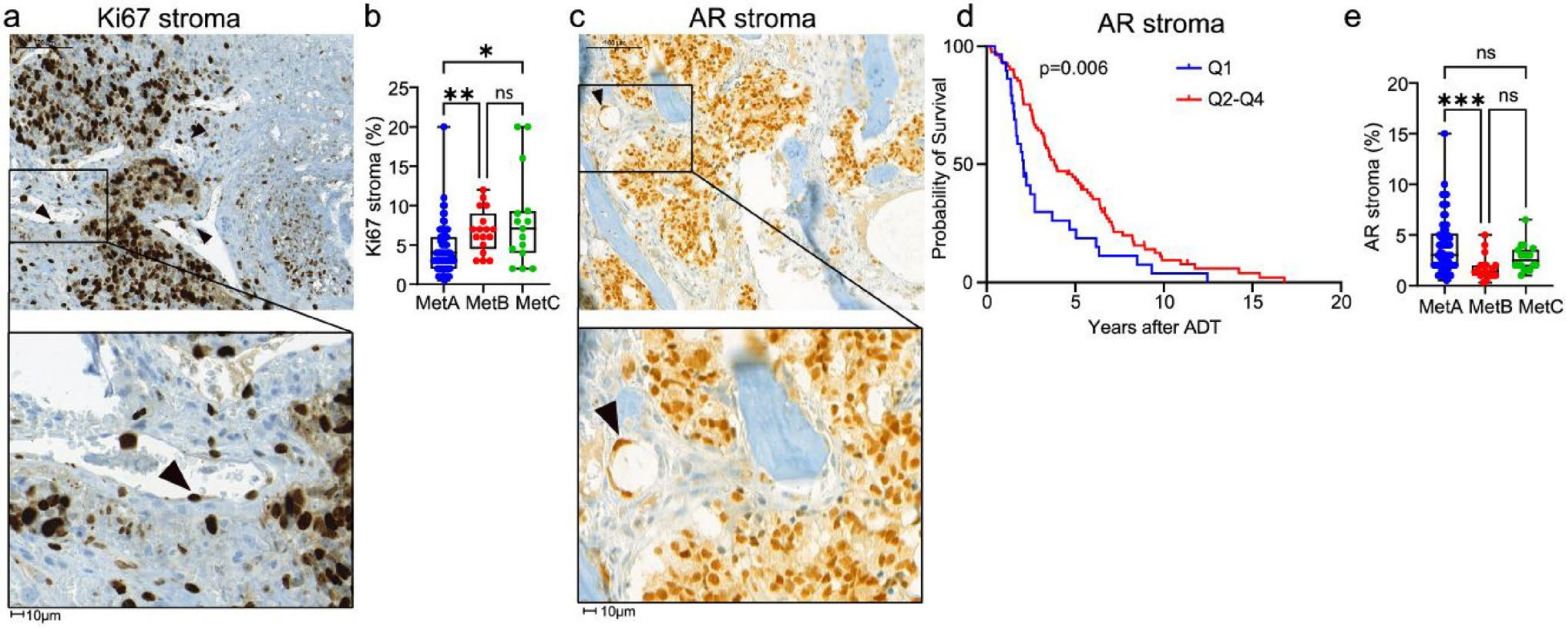
Stroma cell proliferation and androgen receptor expression in relation to the molecular metastasis subtypes MetA-C and patient prognosis. Ki67 (**a**) and AR (**c**) stained sections showing positive immunostaining not only in tumor cells, but also in stroma cells, particularly in blood vessels (arrows). Boxplots showing the lowest density of Ki67 positive stroma cells in MetA (**b**) and the lowest density of AR positive stroma cells in MetB dominated metastases (**e**). Kaplan-Meier analysis showing that survival after androgen-deprivation therapy (ADT) was related to the density of AR + stromal cells (**d**). *p < 0.05, **p < 0.01, ***p < 0.001, ns = not significantly different

SMA + and ERG + stromal cell densities were significantly higher in MetB metastases compared to MetA, while SDF1 + and DCN + cells were significantly lower in MetB metastases (Fig. 2g). No differences were observed in PDGFRB or POSTN densities between metastatic subtypes (Fig. 2g). Higher MetA fraction correlated positively with SDF1 and DCN, and inversely with SMA, while higher MetB fraction correlated positively with SMA and inversely with SDF1 and DCN (Supplement Fig. 2). The MetC fraction did not correlate with any blood vessel or fibroblast markers examined (Supplement Fig. 2). Similarly, PSA score correlated with SDF1 and DCN, and inversely with ERG + endothelium, while tumor Ki67 correlated with SMA and ERG + endothelium (Supplement Fig. 2).

In summary, this suggests that “PSA-high/MetA-enriched” metastases are characterized by lower vascular density and a stroma with higher SDF1 and DCN levels, whereas “Ki67-high/MetB-enriched” metastases have a stroma with higher vascular density and higher SMA levels.

#### Stroma Ki67

To assess reactive stroma, we examined Ki67 expression in stromal cells. The mean fraction of Ki67 + stromal cells was 5% (median 4, Q1 3, Q4 7, range 0.5–20.0, *n* = 127). Ki67 labeling was predominantly observed in endothelial and mural vascular cells (Fig. 3a), suggesting that it mainly reflects vascular proliferation.

The density of Ki67 + stromal cells was not linked to prognosis after ADT (Supplement Fig. 3) but was significantly higher in MetB compared to MetA metastases (Fig. 3b). Accordingly, the MetA fraction and PSA score were inversely correlated with stromal Ki67, whereas MetB fraction and tumor Ki67 positively correlated with stromal Ki67 (Supplement Fig. 2). This indicates that Ki67-high/MetB-enriched metastases have a more reactive stroma with increased vascular proliferation.

#### Stroma androgen receptors

As previously seen, AR staining was prominent in the nuclei of metastatic tumor epithelial cells, while most stromal cells were AR-negative [3, 7] (Supplement Fig. 1 and Fig. 3c). However, low to moderate AR staining was observed in a few osteoblasts, blood vessels, and subsets of inflammatory cells (Fig. 3c), consistent with earlier reports [28, 29]. Only 3% of stromal cells were AR-positive (median 2.7, Q1 1.5, Q4 4, range 0.3–15.0, *n* = 113), demonstrating a major difference from that in primary tumors where stroma cells are mainly AR+ [8].

Survival analysis showed that patients with very low AR + stroma cell density had the shortest survival after ADT (Fig. 3d). MetA metastases had significantly higher stromal AR levels than MetB metastases (Fig. 3e), and AR + stromal cells correlated with MetA fraction and inversely with MetB fraction (Supplement Fig. 2). Similarly, stromal AR correlated with PSA score and inversely with tumor Ki67 (Supplement Fig. 2), suggesting that PSA-high/MetA-enriched metastases have a more androgen-responsive stroma compared to Ki67-high/MetB-enriched metastases.

#### Macrophages and T-lymphocytes

**Fig. 4:**
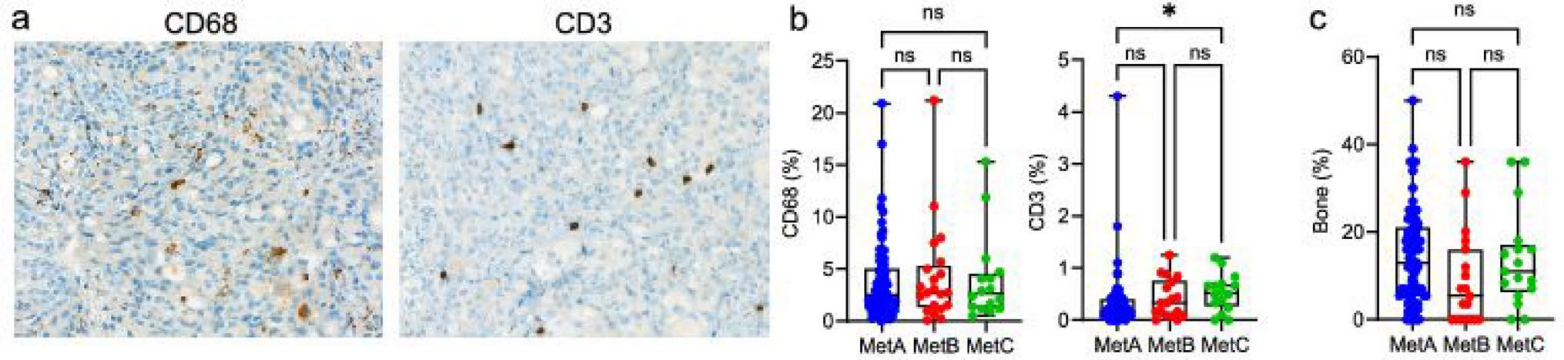
Immune cell infiltration in bone metastases of different molecular subtypes. **a** Sections immunostained for CD68 (macrophages) and CD3 (T-lymphocytes). **b-c)** Boxplots showing densities of CD68, CD3, and bone in relation to dominating metastasis molecular subtypes MetA-C *p < 0.05, ns = not significantly different

To assess immune cell density, we stained the metastases for the pan-macrophage marker CD68 and the T-lymphocyte marker CD3 (Fig. 4a). The mean density of CD68 + macrophages was 4% of the metastasis volume (median 2.6, Q1 1.2, Q4 5.0, range 0–21, *n* = 144), while the mean density of CD3 + T-lymphocytes was 0.4% (median 0.25, Q1 0.10, Q4 0.50, range 0-4.3, *n* = 102). Neither macrophage nor T-lymphocyte densities were associated with survival after ADT (Supplement Fig. 3).

There was no difference in CD68 + cell levels among the MetA-C subtypes, but MetC metastases had significantly higher CD3 + cell levels compared to MetA (Fig. 4b). CD68 showed no association with metastatic subtypes or with PSA or tumor Ki67 but correlated with PDGFRB and CD3 (Supplement Fig. 2). CD3 also inversely correlated with MetA and bone density, but showed no associations with the other subtypes, PSA, or tumor Ki67 (Supplement Fig. 2).

#### Bone

The mean bone density within the metastases was 13%, but varied widely between patients, ranging from 0 to 50% of the metastasis volume (median 11, Q1 5.4, Q4 18, *n* = 140). Interestingly, some “bone metastases” contained very little bone. Bone density was not linked to metastatic subtypes (Fig. 4c) or to ADT outcomes (Supplement Fig. 3). While bone density did not correlate with the MetA-C fractions, it was positively correlated with PSA score and inversely with tumor Ki67 (Supplement Fig. 2), indicating that PSA-high/Ki67 low metastases tend to contain more bone.

### Morphological stroma subtypes

**Fig. 5:**
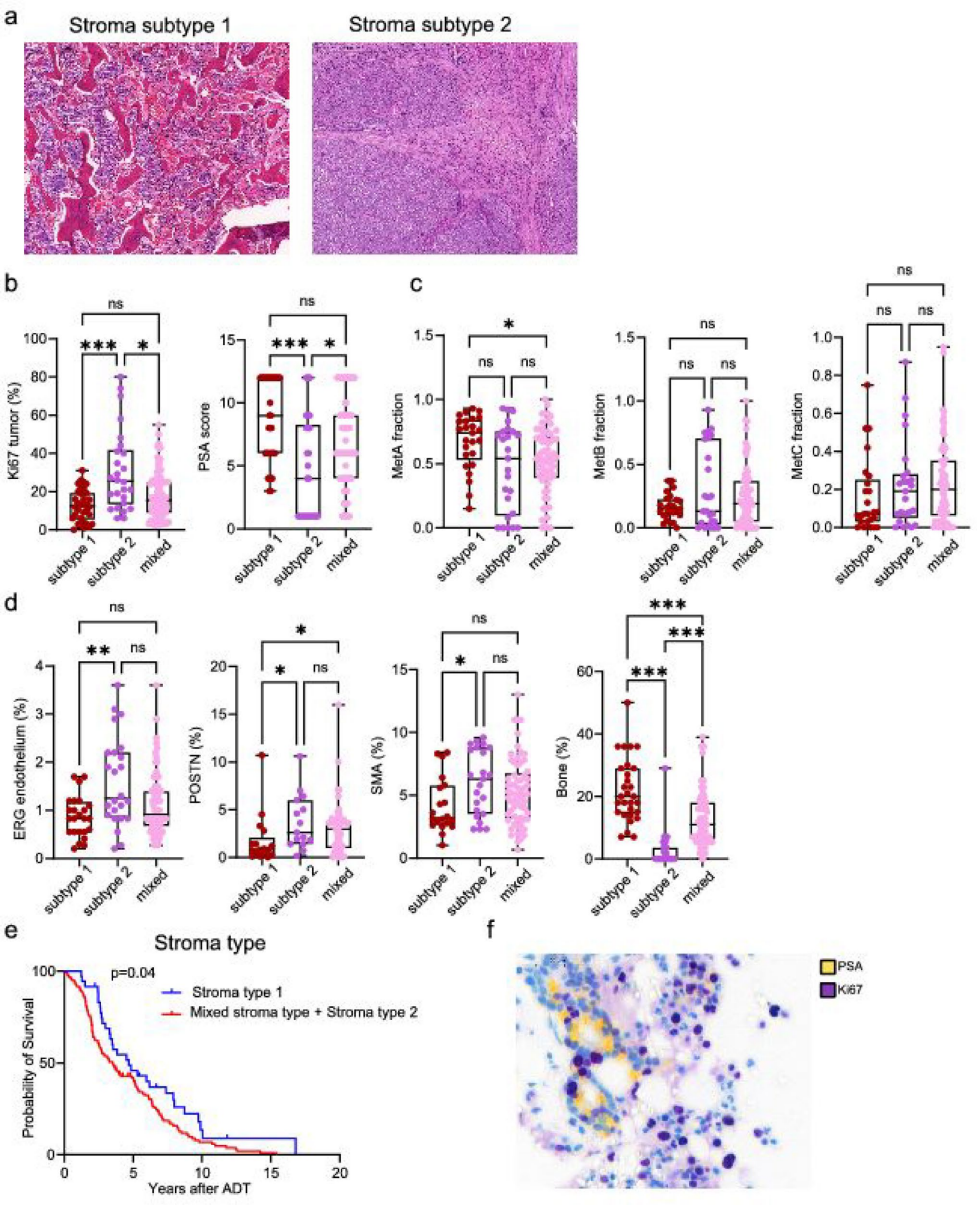
Characteristics of two distinct stoma subtypes in bone metastases from prostate cancer. **a** Htx-eosin-stained sections representing stroma subtype 1 (tumor cells growing in stroma spaces separated by bone-tissue), and stroma subtype 2 (tumor cells growing in a prominent connective tissue stroma). Boxplots showing **b** Ki67 and PSA staining, **c** metastasis fractions of cells with MetA-C characteristics, and **d** densities of ERG, POSTN, SMA, and bone in relation to metastases stroma subtypes (type1, type 2 or mixed type1-type2). *p < 0.05, **p < 0.01, ***p < 0.001, ns = not significantly different. **e** Kaplan-Meier analysis showing that stroma subtypes are related to survival after androgen-deprivation therapy (ADT). **f** Section of a micro-metastasis double stained for PSA + Ki67, showing tumor epithelial cells forming gland like, PSA positive structures growing in the bone marrow within a stroma subtype type 1

We also analyzed the general morphology of the bone metastases and identified three distinct stroma subtypes: type 1 (*n* = 36), type 2 (*n* = 26) and mixed type (*n* = 96) metastases. The type 1 metastases had nests of tumor epithelial cells growing in spaces separated by bone tissue, and by thin strands of stroma containing small blood vessels (Fig. 5a). The tumor cells were closely associated with each other, bone surfaces, and remaining bone marrow components, but contained few fibroblast-like cells and no smooth muscle cells. In contrast, the type 2 metastases contained little bone or bone marrow, with tumor cells growing in a vascularized stroma composed of fibroblast-like cells, extracellular matrix, and some inflammatory cells (Fig. 5a). The stroma formed septa that separated tumor cell nests, varying in thickness and vascularity. Tumor cells were not directly in contact with bone or hematopoietic cells but were closely associated with other tumor cells, endothelial cells, pericytes, and fibroblast-like cells. The largest group of mixed type metastases exhibited type 1 and type 2 areas in different parts of the same metastasis, indicating that spatial heterogeneity is very common and perhaps underestimated due to insufficient sampling from all parts of a metastasis.

Type 2 metastases had significantly higher tumor cell proliferation and lower PSA score compared to type 1 and mixed metastases (Fig. 5b), but the fractions of MetA-C were not related to stroma subtype (Fig. 5c). Among the stroma factors, ERG + endothelial cells, POSTN levels, and SMA levels were higher in type 2 metastases, while bone density was lower (Fig. 5d). Type 1 metastases were significantly associated with better prognosis after ADT compared to mixed and type 2 metastases (Fig. 5e).

In some patients with adjacent vertebral tissue sampled, we identified micro-metastases consisting of small tumor cell clusters located on bone surfaces or within normal bone marrow near blood vessels and hematopoietic cells, classifying as type 1 metastases (Fig. 5f). In this study we did not quantify epithelial and stroma markers in micro-metastases further.

We then compared the morphology of metastases from operated cases (spine and hip) and from biopsy samples (crista and rib). Among the factors measured, we found that the density of CD68 + macrophages was significantly higher in the operated cases in comparison to biopsied cases (4.1 ± 3.9% vs. 0.64 ± 0.66%, *n* = 132 and *n* = 12, respectively, *p* < 0.001). Moreover, Ki67 + cells in the stroma and MetC fraction were significantly higher in spine and hip compared to crista (Ki67 stroma: 5.6 ± 4.2% vs. 2.9 ± 1.4%, *n* = 118 and *n* = 9, respectively, *p* = 0.019, MetC: 0.23 ± 0.22 vs. 0.14 ± 0.19, *n* = 111 and *n* = 20, *p* = 0.021).

In summary, metastases with type 1 stroma were associated with higher bone content, lower tumor cell Ki67 levels, higher PSA scores and slightly better prognosis. In contrast, metastases with type 2 stroma contained less bone and had more ERG + blood vessels and POSTN + cells, exhibiting higher tumor proliferation and lower PSA levels.

### Epithelial and stromal factors in relation to treatment

**Fig. 6:**
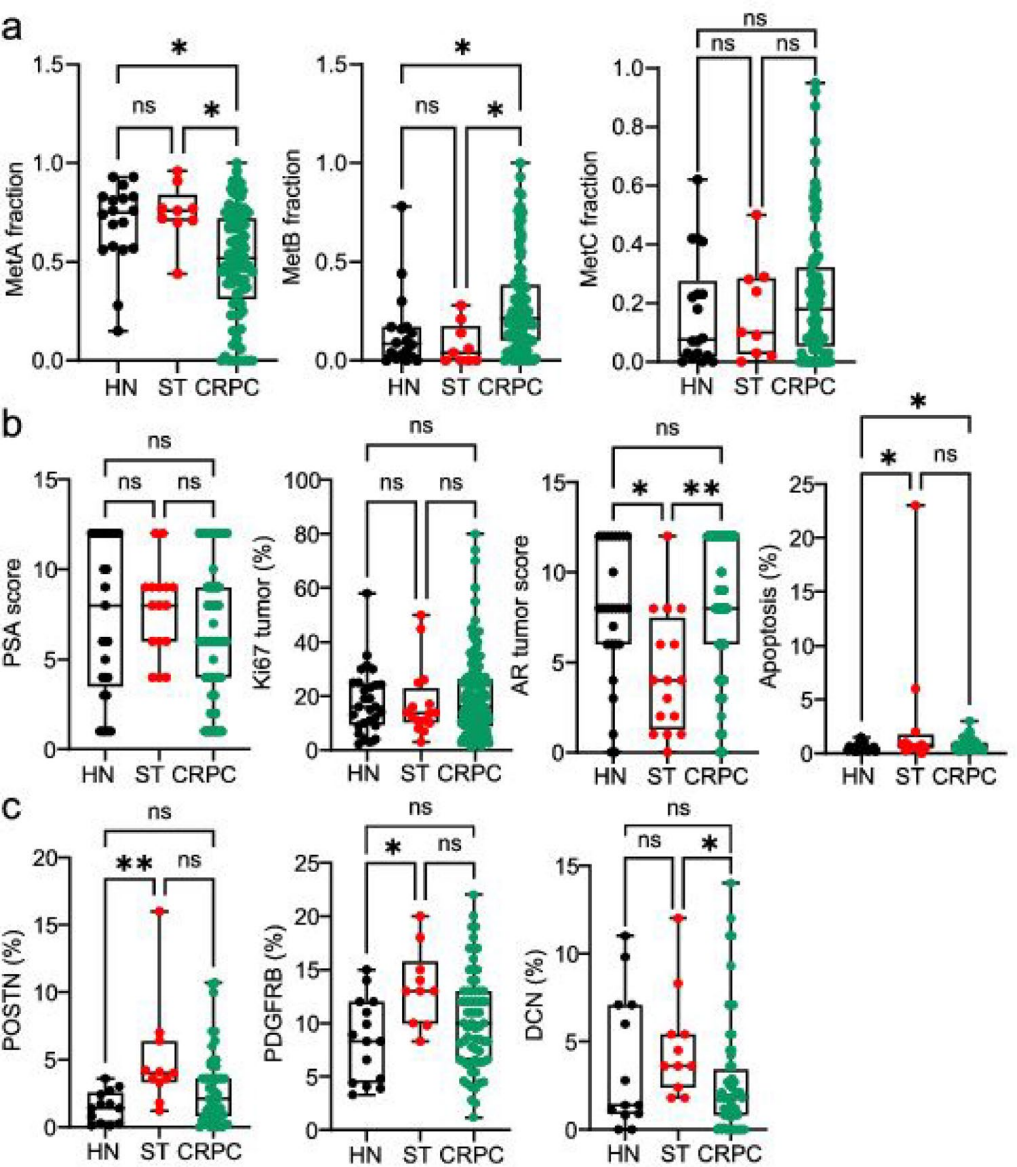
Molecular metastasis subtypes and cellular markers in relation to hormone therapy. Boxplots showing metastasis fractions of cells with MetA-C (**a**), PSA score, Ki67, AR tumor score, and apoptosis (**b**), and densities of POSTN, PDGFRB, and DCN (**c**) in patients with hormone-naïve (HN), short-term androgen-deprivation therapy (ST), and castration resistant prostate cancer (CRPC). *p < 0.05, **p < 0.01, ns = not significantly different

In this study we had access to metastases from 30 hormone-naïve, 16 short-term ADT, and 121 CRPC patients. We found that the MetA fraction was lower in CRPC compared to hormone-naïve metastases, while the MetB fraction was higher in CRPC relative to the other cases (Fig. 6a). There were no significant changes in PSA score or Ki67 levels among hormone-naïve, short-term treated, or CRPC patients; however, short-term treatment appeared to lower tumor epithelial AR levels and to increase apoptosis (Fig. 6b). Also, apoptosis was slightly higher in CRPC compared to hormone-naive cases (Fig. 6b).

Among the stromal factors examined, POSTN and PDGFRB were significantly higher in metastases from short-term ADT patients compared to hormone-naïve patients, while DCN levels were significantly lower in CRPC patients compared to those receiving short-term treatment (Fig. 6c).

In addition to ADT, some patients had received therapies for CRPC (Table 1). In this cohort, bicalutamide treatment was associated with longer survival, while 2nd and 3rd line therapies and/or palliative radiation therapy towards the operated vertebra showed non-significant tendencies to increase survival (Supplementary Fig. 4). Metastases that had been radiated showed higher bone density and stroma AR staining than the rest, and previous bicalutamide treatment was associated with higher epithelial AR and stroma SMA staining (Supplement Fig. 4). Chemotherapy showed no obvious relation to any of the morphological markers examined (Supplement Fig. 4). For abiraterone and other second or third-line therapies for CRPC the number of men treated was too low for morphological comparisons (Table 1).

In summary, short-term effects of ADT were observed on tumor cell nuclear AR levels and apoptosis, as well as on stromal POSTN and PDGFRB levels. CRPC metastases had higher tumor cell apoptosis compared to untreated patients and were enriched for the MetB subtype. Moreover, bicalutamide treatment for CRPC affected epithelial AR and stroma SMA levels.

## Discussion

PC bone metastases can be divided into different subgroups, based on morphological and/or molecular diversities [6, 7, 9, 13, 14, 30]. In this study, we extend these observations by investigating morphological heterogeneities within individual metastases and between patients and examining how these variations relate to molecular tumor characteristics and patient prognosis after ADT.

As earlier reported [6, 7], and now confirmed in a larger cohort, we show that metastases with high proliferation and poor differentiation (Ki67 high, PSA low, MetB-enriched) are related to a particularly bad outcome after ADT. These metastases have low bone density (stroma subtype 2), high endothelial cell density, high stroma proliferation, high density of SMA, but low stroma AR density, and low densities of DCN and SDF-1. In contrast, metastases with lower proliferation, higher differentiation, and better outcome after ADT (Ki67 low, PSA high, MetA-enriched), often grow in more normal bone structures (stroma subtype 1). These cases also have higher stroma levels of AR, DCN, SDF-1, and lower endothelial cell density.

As previously demonstrated, cell differentiation and growth in normal prostate and primary prostate tumors are influenced by bidirectional communication between epithelial cells and the surrounding stroma [21–23, 31–33, 34]. Here, markers like Ki67 and PSA in metastatic tumor cells could be linked to cellular diversities in the metastatic stroma suggesting that stroma cells play an important role in controlling tumor cell proliferation and differentiation also in metastases. The response to ADT in primary tumors is, in addition to direct effects on epithelial cells, also dependent on effects in AR + stroma cells [21, 35], with loss of AR + smooth muscle cells in the prostate stroma being linked to a weak ADT response in patients [36]. In line with this, we here found that lower AR levels in metastatic stroma were associated with poorer outcomes after ADT. Notably, AR levels were higher in Ki67-low/PSA-high/MetA metastases compared to Ki67-high/PSA-low/MetB metastases, suggesting that stroma androgen responses differ between these subtypes.

Short-term hormonal treatment appeared to reduce tumor cell AR staining and increase tumor cell apoptosis in bone metastases, while leaving cell proliferation (Ki67) and PSA expression unaffected. This contrasts with primary tumor tissue, where reduced AR staining is accompanied with a clear reduction in tumor cell proliferation and moderately increased apoptosis during the first weeks after surgical castration [4]. Thus, it is likely that the bone-environment acts to reduce the acute effects of ADT on tumor cells (see also [2]). As demonstrated in an experimental rat model for PC bone metastases, high IGF-1 levels within the bone environment may counteract effects of castration in PC metastases and, furthermore, IGF receptor inhibition could possibly be used to strengthen the ADT response in bone metastases [37].

When PC cells reach the bone marrow, they first colonize the hematopoietic stem cell niche at the bone surface and around blood vessels [38–43]. At this early metastasis stage, the tumor microenvironment includes various cell types like osteoblasts, osteoclasts, endothelial cells, pericytes, adipocytes and inflammatory cells [40, 42]. Accordingly, we noted this type of microenvironment in micro-metastases and in areas of metastases with a bone-rich stroma type 1. Presumably, as the metastasis grows, tumor cells fill up the bone marrow spaces and replace the bone and bone marrow, finally spreading outside the bone. Some metastases in our study (primarily those with low Ki67/high PSA/MetA) seemed to retain or form new bone structures (stroma type 1), while in others (mainly in those with high Ki67/low PSA/MetB) the bone and bone marrow were replaced by a more connective tissue-like microenvironment (stroma type 2). In line with this, patients with stroma type 1 had slightly better outcome after ADT. As most, and perhaps all, contained both type 1 and type 2 areas treatment responses probably vary between different parts of the same metastasis.

Most studies on how the bone metastasis microenvironment influences PC dormancy and metastasis growth have been performed in vitro or in animal models and mainly focused on how tumor cells interact with bone cells like osteoblasts and osteoclasts and stem cell-niches [40, 42, 44–49]. In line with this, bone metastases are commonly classified as osteoblastic, osteolytic or mixed [30, 40, 42, 48], stressing the importance of bone cells in metastasis biology. However, our findings, in line with Roudier et al. [14, 30], indicate that in advanced clinical bone metastases, the presence of bone and bone marrow is limited and, thus, few cancer cells are located near bone surfaces. This could explain why therapies targeting bone-tumor interactions in advanced patients have limited success [50]. Consequently, the vicious cycle between bone cells and PC cells [42, 48] likely plays a larger role during bone metastasis establishment than in later phases. In clinically established metastases, we showed that most tumor cells were clustered around small blood vessels (ERG+, SDF1+, POSTN+, SMA+, PDGFRB+), suggesting that other cell interactions, especially involving blood vessels, might drive later stages of metastasis growth.

Consistent with this, the density of endothelial cell ERG + nuclei was positively correlated with cell proliferation and negatively correlated with cellular PSA levels, and higher blood vessel density was associated with a poorer outcome after ADT. The proximity of blood vessels to tumor epithelial cells suggests a possible role for angiocrine signaling or angiogenesis in regulating metastasis growth and treatment responses [39, 51]. For instance, endothelial cells can protect metastatic cancer cells from cytotoxic drugs [38]. Moreover, in normal prostate tissue, castration-induced gland involution is preceded by vascular involution and a significant drop in blood flow, and testosterone-induced glandular regrowth follows VEGF-mediated vascular growth in castrated animals [52, 53]. In the current study, however, ADT did not affect endothelial cell density in bone metastases. The importance of blood vessels in the response to ADT in PC bone metastases remains unclear.

Many studies in primary tumors have focused on the various stimulatory and inhibitory effects of different types of cancer-associated fibroblasts (CAFs) [19, 20, 23, 54–56]. While CAFs are prevalent in primary prostate tumors, they are less abundant but not absent in bone metastases [8]. In this study, we identified fibroblast-like cells, particularly in metastases with a type 2 stroma. Baryawno et al. [25] demonstrated that bone metastases can contain up to 17 different non-bone stromal cell types. Together with the findings from our study, which also shows the importance of various non-bone stromal cells, this highlights the need for further research to better understand their roles and impact.

Factors like SDF-1, POSTN, SMA and PDGFRB were found both in blood vessels and in fibroblast-like cells. SDF1 (also known as CXCL12) binds to receptors on PC cells and thereby plays a role in tumor-cell homing to the bone marrow [42]. Additionally, SDF-1 is involved in inducing metastasis dormancy [57]. Our findings showed high SDF-1 levels in MetA cases, compared to the other metastatic subtypes. POSTN, located in blood vessels and fibroblast-like cells, was higher in metastases with a mixed or type 2 stroma and the only stromal factor examined here that was coupled to a worse prognosis without being related to the Ki67 high/PSA low/MetB subtype. In the normal prostate, POSTN is primarily expressed in the stroma, particularly in endothelial cells, and increased expression in primary tumors is related to enhanced aggressiveness [58, 59]. Endothelial cells in newly formed blood vessels within tumors release POSTN, which promote the outgrowth of bone metastases in experimental models [39]. Additional studies are needed to clarify the specific role of POSTN in PC bone metastasis. PDGFRB staining in the stroma did not correlate with outcomes or metastasis molecular subtypes, but we cannot exclude the possibility that spatial differences, i.e. expression in blood vessels penetrating tumor cell nests, could give different effects than PDGFRB positive cells more remote from tumor epithelial cells.

DCN expression was mainly found in fibroblast-like cells and in the matrix in the bone metastasis stroma and the current study suggests that reduced synthesis of DCN may contribute to the growth of the most aggressive bone metastases (Ki67 high, PSA low, MetB). In line with this, lower DCN levels were associated with a worse outcome. DCN plays a crucial regulatory role in normal prostate tissue and primary PC. It is typically expressed in fibroblast-like cells adjacent to prostate glands but is down-regulated in higher-grade tumors [60, 61]. DCN is also expressed in osteoblasts and in all five fibroblast-like cell types present in normal bone marrow [25]. It promotes epithelial cell differentiation and inhibits proliferation [62], and in line with this correlated with tumor PSA levels in the metastases. Additionally, DCN inhibits several key receptors involved in prostate cell functions, such as AR, EGFR, TGFBR, IGFR, MET and PDGFR [63, 64]. DCN also suppresses angiogenesis, tumor growth, and bone metastasis in PC models [63–67]. In this study, DCN was found to be absent to low in the small stromal septa near tumor cell nests, especially in MetB cases. Given its potential therapeutic role [64, 66] testing DCN as a novel treatment for MetB could be of interest. Hypothetically, the combination of low SDF-1 and DCN levels, along with elevated local PDGFRB expression, may induce changes in both the vasculature and non-vascular components of the stroma, leading to a more aggressive bone metastasis phenotype.

The growth of PC cells in the bone marrow leads to an altered immune microenvironment [26] and various immune cell types may also affect how PC cells respond to ADT [68–70]. In our study, quantification of CD68 + macrophages and CD3 + lymphocytes did not reveal any clear association between the level of immune cell infiltration and treatment response. Also, we observed no differences in immune cell infiltration between metastases of different molecular subtypes. However, the possibility that tumor infiltration of different monocyte and T cell lineages may be involved in regulating tumor response to ADT as well as to other therapies, including immunotherapy, needs to be further investigated.

Importantly, most tumor epithelial cells in the clinically manifested bone metastases were not located next to stroma cells, rather, they were located close to other tumor epithelial cells, exhibiting variations in epithelial cell phenotypes. Molecular subtype analysis suggests that most metastases contain a mix of cells with MetA-C characteristics [6]. IHC markers related to MetA and B subtypes (PSA and Ki67) had significant intra-metastasis heterogeneity. Furthermore, heterogeneities were also found for stroma subtype 1 and 2, suggesting also stromal heterogeneity in metastases, similar to what has been previously described for primary PC [20, 54, 56, 71–73]. This suggests that many metastases contain mixtures of tumor cell subclones with different phenotypic characteristics, furthermore, being in contact with local variations in the microenvironment. If interactions among different subclones of tumor epithelial cells affect metastasis behavior and treatment responses, and if treatment effects vary within different regions of the same metastasis, and between different metastases, this needs to be considered when designing optimal treatment protocols.

In summary, the current study verifies that high tumor cell proliferation and low PSA expression in PC bone metastases are clearly related to an aggressive molecular subtype (MetB) and poor patient prognosis after ADT. The study also demonstrates the relationship between this aggressive metastasis phenotype and morphological characteristics of the bone microenvironment (high endothelial density, high POSTN and SMA expression as well as low DCN expression). Importantly, our observations suggest that PC bone metastases, like primary prostate tumors and other tumor types [74], show high inter and intra metastatic heterogeneity, harboring different tumor subclones interacting with each other and with various stromal cells, like endothelial cells, pericytes and fibroblasts. The role of those stroma cells, particularly of those in the blood vessel walls, remains unclear and needs to be examined in more detail in relation to clonal response and treatment resistance, for example by using single-cell and spatial transcriptomics on human metastasis samples. Such studies could uncover critical insights into metastasis stroma heterogeneities and their importance for growth and treatment responses in different metastasis molecular subgroups and in different parts of a single metastasis.

## Electronic supplementary material

Below is the link to the electronic supplementary material.

Supplementary Material 1

## Data Availability

No datasets were generated or analysed during the current study.
