# Supplementary material for: Morphological heterogeneities in prostate cancer bone metastases are related to molecular subtypes and prognosis
Source: Clin Exp Metastasis. 2025 Aug 21;42(5):49. doi: 10.1007/s10585-025-10365-y (PMC12370827; doi:10.1007/s10585-025-10365-y)
Supplement: Supplementary file 1 — Supplementary Material 1 [file 10585_2025_10365_MOESM1_ESM.pdf]

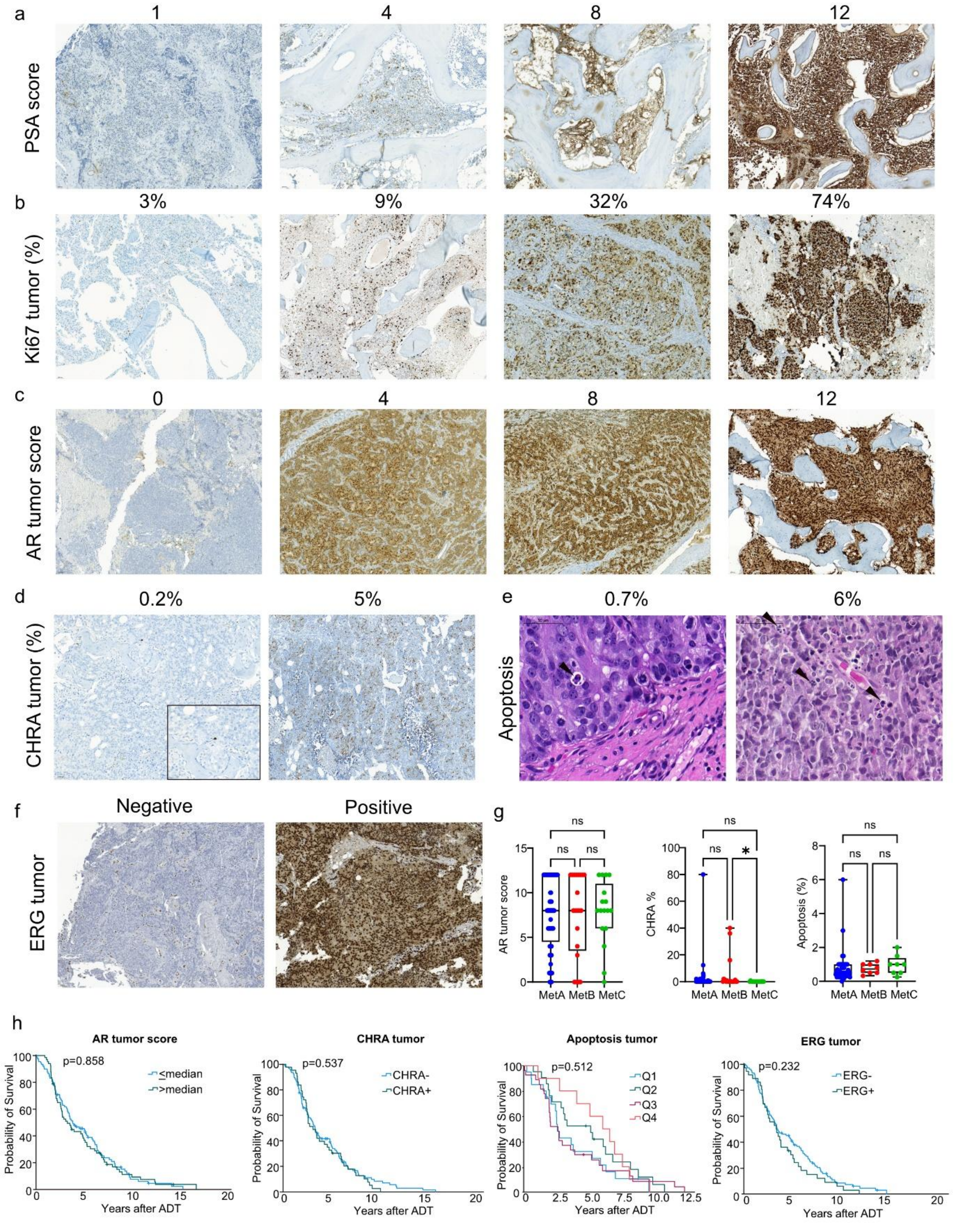

Supplement Figure 1: **Morphological heterogeneities in prostate cancer bone metastases and epithelial markers showing no clear relations to the molecular metastasis subtypes MetA-C or patient prognosis.** Examples of **a**) PSA-stained metastases with different staining scores, **b**) Ki67-stained metastases with different fractions of stained tumor cells, **c**) AR-stained metastases with different epithelial staining scores, **d**) CHRA stained metastases with different fractions of CHRA-positive cells (insert in higher magnification of a single CHRA-positive cell surrounded by negative cells), **e**) Htx-eosin-stained metastasis sections with different fractions of apoptotic cells (arrow-marked) and **f**) ERG-stained metastases showing that some tumor cells were ERG-positive whereas others were negative. **g**) Boxplots

of tumor epithelial cell AR-staining score, and the fractions of CHRA-positive, and apoptotic tumor epithelial cells in dominating metastasis subtypes MetA-C. *p<0.05, ns=not significantly different. **h**) Kaplan-Meier plots showing that tumor epithelial AR, CHRA, apoptosis and ERG in bone metastases were not associated with survival after ADT.

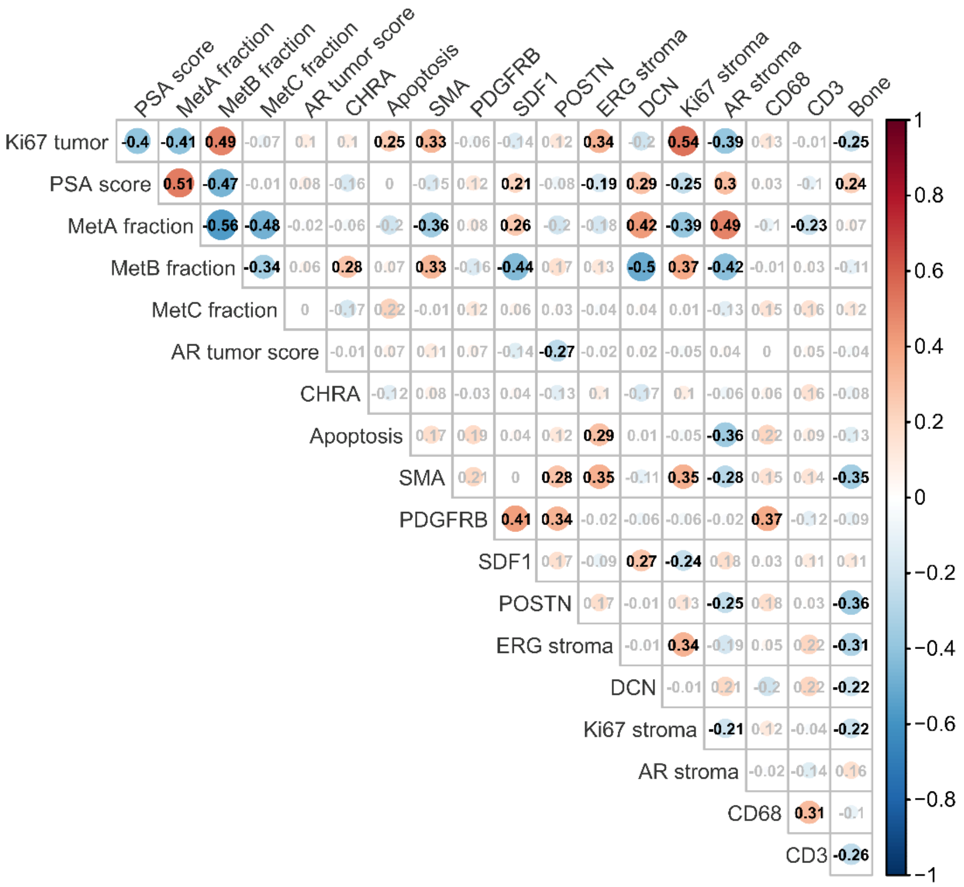

Supplement Figure 2: **Spearman's Rho correlations**. Significant correlations (positive; red, negative; blue) are shown in bold (p<0.05).

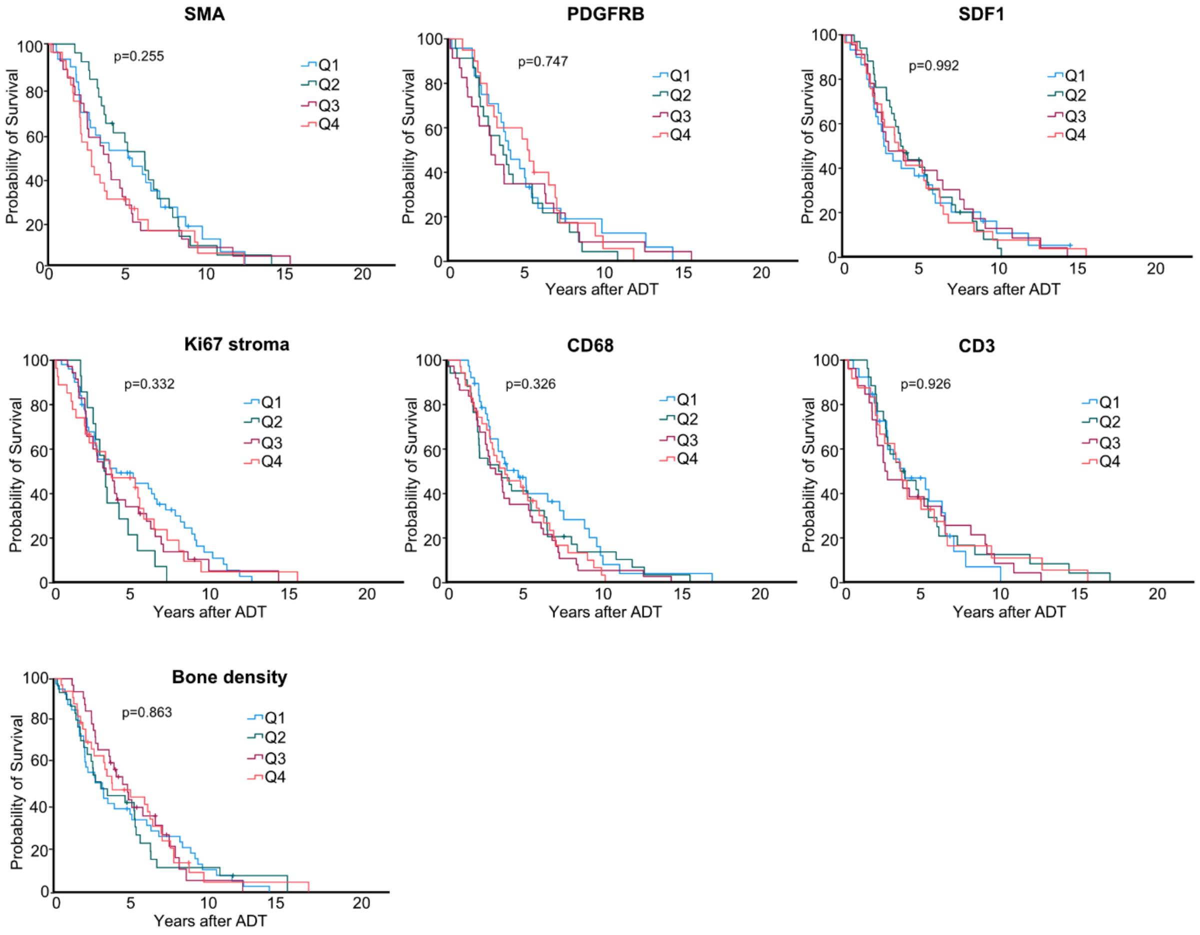

Supplement Figure 3: **Stromal markers in prostate cancer bone metastases showing no clear relations to patient prognosis**. Kaplan-Meier curves that demonstrate that the densities of stroma markers SMA, PDGFRB, SDF1, Ki67, CD68, CD3, and bone were unrelated to outcome after androgen-deprivation therapy (ADT).

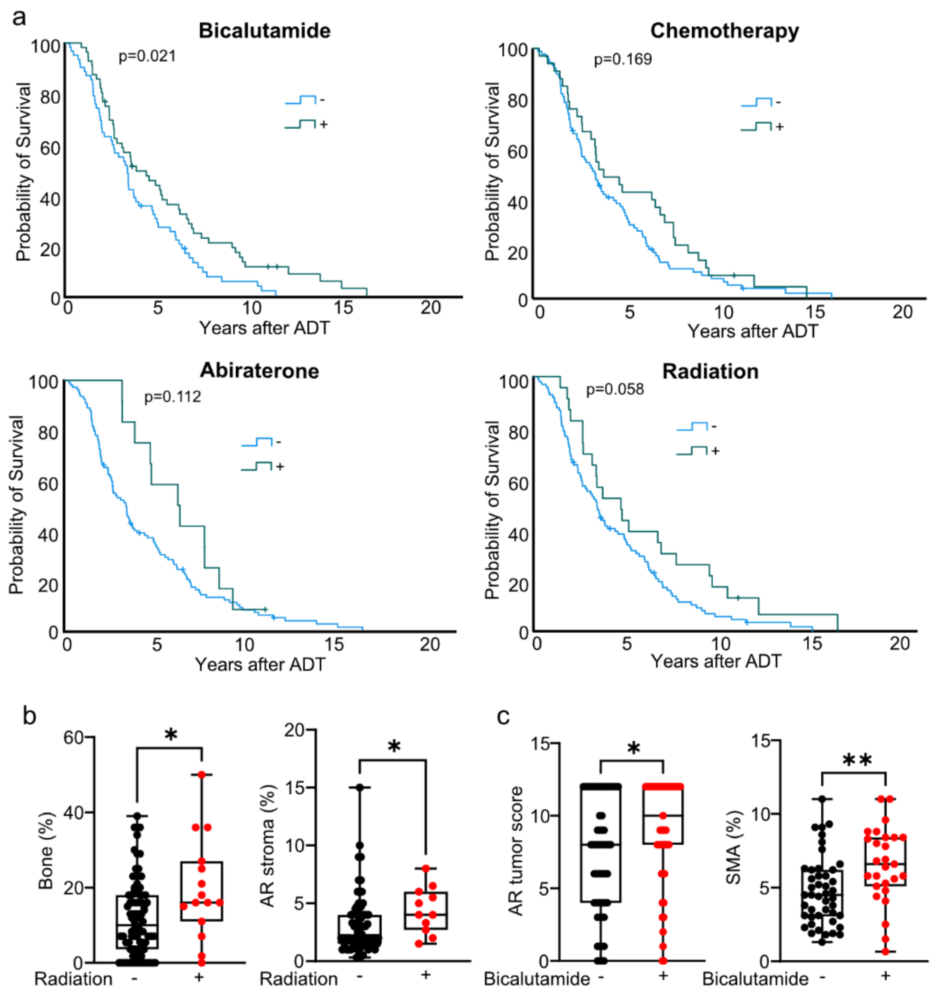

Supplement Figure 4: **Additional treatments to androgen-deprivation therapy in castration-resistant patients**. **a**) Kaplan-Meier curves demonstrate that previous bicalutamide treatment significantly improved survival, while chemotherapy, abiraterone, and radiation towards the operated vertebra tended to improve survival in this patient cohort. **b**) Radiation was associated with higher bone density and AR stroma levels and **c**) bicalutamide was associated with higher AR tumor score and higher levels of SMA.
